# Dimensions of sexual orientation and sleep disturbance among young adults

**DOI:** 10.1016/j.pmedr.2017.07.008

**Published:** 2017-08-05

**Authors:** Julie Fricke, Maria Sironi

**Affiliations:** a Department of Health and Behavioral Sciences, University of Colorado Denver, North Classroom Building, Room 3018, Denver, CO, 80217, United States; b Department of Social Science, University College London, 20 Bedford Way, London, WC1H 0AL, United Kingdom

## Abstract

We examined associations among 3 dimensions of sexual orientation (identity, attraction, and behavior) and sleep disturbance among young adults in the United States. Using Wave IV of the National Longitudinal Study of Adolescent Health (respondents aged 24–32, N = 14,334), we ran multivariate logistic regressions to estimate the probability of reporting trouble falling asleep, trouble staying asleep, and short sleep duration, based on specific sexual orientation categories.

Results after controlling for mental health indicate that these categories are more likely to have trouble falling asleep: women who identify as “bisexual” (OR = 1.85, CI: 1.21,2.82), women attracted to “both sexes” (OR = 1.31, CI: 1.00,1.72), women who have had “mostly opposite sex” partners (OR = 1.40, CI: 1.10,1.77), and men who have had “mostly same sex” partners (OR = 2.28, CI: 1.21,4.31). For trouble staying asleep: women who identify as “bisexual” (OR = 1.48, CI: 1.01,2.18), men and women attracted to “both sexes” (OR = 1.81, CI: 1.12,2.91; OR = 1.27, CI: 1.00,1.60), and women who have had “mostly opposite sex partners” (OR = 1.38, CI: 1.13,1.69). For short sleep duration: women who identify as “mostly straight” or “mostly gay” (OR = 1.27, CI: 1.01,1.60; OR = 2.64, CI: 1.36,5.14), men who identify as “bisexual” (OR = 2.56, CI: 1.26,5.18), women attracted only to “same sex” (OR = 2.42, CI: 1.48,3.96), men attracted to “both sexes” (OR = 1.88, CI: 1.21,2.93), and women who have had “mostly same sex” partners (OR = 4.90, CI: 2.10,11.46). Given the variation in findings, it is necessary to analyze each sexual orientation dimension and the categories within each dimension to adequately understand sleep disturbances among sexual minority populations.

## Highlights

• Sleep disturbances across sexual identity, sexual attraction, and sexual behavior are analyzed.
• Specific intermediate categories of sexual orientation are more at-risk for sleep disturbances.
• Mental health helps to explain sleep disturbances among sexual minority men more than among women.

## 1 Introduction

Conceptually, sexual orientation has three major dimensions (Badgett and Goldberg, 2009): sexual identity, sexual attraction, and sexual behavior; however, past work linking sexual orientation to health has often failed to recognize this multidimensionality. Notably, this may stem from lack of available data, as few large-scale surveys include questions regarding each dimension. Of this past work, most have found sexual minority status to be disadvantageous to both mental and physical health (Meyer and Northridge, 2007), which is commonly explained by the minority stress model (Meyer, 2003). Reliance upon a single dimension of sexual orientation misses important variations in health behaviors (McCabe, Hughes, Bostwick, West, and Boyd, 2009), and leaves us with a deficient understanding of disparities not only between sexual minority and majority populations, but also within sexual minority populations themselves (Lindley, Walsemann, and Carter, 2012). The way in which sexual orientation is defined and measured has important implications for health research and practice (McCabe, Hughes, Bostwick, and Boyd, 2005).

Findings from studies that have analyzed all three dimensions of sexual orientation have been consistent with the aforementioned literature documenting poorer health outcomes for sexual minority populations as compared to heterosexuals (Meyer and Northridge, 2007), but they have also shown considerable variability across dimensions and genders in relation to health-risks (McCabe et al., 2009, Lindley et al., 2012, McCabe et al., 2005, McCabe et al., 2013, Talley et al., 2015, Lhomond et al., 2014, Brewster and Tillman, 2012, Bostwick et al., 2010). For example, Lindley et al. (Lindley et al., 2012) utilized the National Longitudinal Study of Adolescent Health ‘Add Health’ to assess the dimensions of sexual orientation and associations with common health-risks experienced by sexual minorities. Notably, the Add Health dataset allows for an analysis of intermediate sexual orientation categories (e.g. “mostly straight,” “mostly opposite-sex partners”), which, as the authors mention, are preferred, because they better reflect personal experiences (Lindley et al., 2012). The results showed that among women aged 24–32 years, being attracted to both sexes, identifying as “mostly straight” or “bisexual,” and having “mostly opposite-sex sexual partners” was associated with greater risk of depressive symptoms, perceived stress, smoking, binge drinking, and victimization. While among men of the same age, identifying as “mostly straight” was associated with a greater risk for depressive symptoms, perceived stress, and smoking. Such variation across dimensions calls for the analysis of all three whenever possible (McCabe et al., 2005).

Potential differences regarding sleep behavior, a key indicator of long-term health (Åkerstedt, 2006) and a growing public health concern (Colten, Altevogt, and Colten, 2006), have yet to be studied across dimensions of sexual orientation. A recently published article by Chen and Shiu (Chen and Cheng-Shi, 2016) examined the association between sexual orientation and sleep disturbance among adults aged 20 and older in the U.S. using the 2013–2014 National Health Interview Survey. The authors found that sexual minority adults had higher risks of sleep disturbances (trouble falling asleep, trouble staying asleep, not feeling rested) and short sleep duration than heterosexual adults, which was partly explained by differences in socioeconomic status and physical and mental health conditions. Specifically, sexual minority women had greater odds of waking up at night than sexual minority men, but sexual orientation was not associated with an increased risk of short or long sleep duration. However, the authors only incorporated sexual identity as an indicator of sexual orientation, distinguishing between heterosexual, homosexual, bisexual, and “other” minority individuals. Because the authors are unable to include the other dimensions of sexual orientation, the study is not sufficient to understand possible differential effects of sexual orientation on sleep behavior.

Previous work has also demonstrated that inconsistency among dimensions is fairly common (Badgett and Goldberg, 2009, Lindley et al., 2012), which may further contribute to health-risks for individuals that express diverse and fluid sexual identities, attraction, and behavior (Talley et al., 2015, Lourie and Needham, 2016). Discordance regarding sexual orientation is more pertinent among young adults (Strutz, Herring, and Halpern, 2015), as sexual orientation in youth is not fixed (Diamond, 2000, Rosario et al., 2006), especially among females (Ott, Corliss, Wypij, Rosario, and Austin, 2011), with variation in the timing and sequence of orientation indicator affirmation through young adulthood (Rosario, Schrimshaw, and Hunter, 2008). Further, early adulthood is often accompanied by greater anxiety and uncertainty because of transitions and role formations (Mirowsky and Ross, 1992). Therefore, a comprehensive examination of sexual orientation in regards to young adult health-risks is of particular necessity.

Thus, this work utilizes Wave IV of the Add Health dataset, a nationally representative sample of young adults, to examine the three dimensions of sexual orientation and their associations with sleep disturbance. We also test whether the associations change after controlling for mental health, as sexual minorities report worse mental health outcomes than the majority (Meyer and Northridge, 2007), and poor mental health is strongly related to impaired sleep (Åkerstedt, 2006, Gillin et al., 1984, Chapman et al., 2013, Spoormaker and Van Den Bout, 2005).

## 2 Methods

For this research, we used Wave IV [restricted use] of Add Health. Add Health is a longitudinal study of a nationally representative sample of adolescents in grades 7–12 in the United States during the 1994–1995 school year. The Add Health cohort has been followed into young adulthood with four in-home interviews, the most recent in 2008, when the sample was aged 24–32.

We began with 15,701 individuals, and then restricted the analyses to respondents that were assigned a probability weight (14,800) at Wave IV to ensure that the sample was representative of specific age groups. Further, we excluded 466 individuals who did not have information on the variables included. Our final sample consisted of 7646 women and 6688 men (14,334).

### 2.1 Included variables

We used three dependent variables in this analysis regarding sleep disturbance, measured at Wave IV: trouble falling asleep, trouble staying asleep, and short sleep duration (< 6 h per night) on a typical work day. Trouble falling asleep and trouble staying asleep are categorical variables based on the questions, “Over the past four weeks, how often did you have trouble falling asleep?” and “Over the past four weeks, how often did you have trouble staying asleep throughout the night?” Categories for both questions include “never,” “less than once a week,” “1 or 2 times a week,” “3 or 4 times a week,” and “5 or more times a week.” Since we are interested in chronic sleep problems, we recoded both trouble falling and staying asleep into dichotomous variables: “had trouble falling (or staying) asleep 3 or 4 times a week or more in the past four weeks” (Badgett and Goldberg, 2009) and “had trouble falling (or staying) asleep 1 or 2 times a week or less in the past four weeks” (0). We computed short sleep duration by combining the variables of usual time falling asleep and usual time waking up during the week on a typical work day (respondents reported clock-time indicating AM/PM), and then recoded it into a dichotomous variable: “sleep less than 6 hours on a typical work day” (Badgett and Goldberg, 2009) and “sleep 6 or more hours on a typical work day” (0).

In order to take into account all the three dimensions of sexual orientation – identity, attraction, and behavior –, we used three different independent variables in our analysis, also measured at Wave IV. The first is *sexual identity*, a categorical variable based on the question, “Please choose the description that best fits how you think about yourself.” The categories given are “straight,” “mostly straight,” “bisexual,” “mostly gay,” “gay,” and “none.” The second is *sexual attraction*, a categorical variable that we constructed based on the gender of the respondent and their reported romantic attraction(s). Each respondent is asked if he or she is “romantically attracted to females,” and if he or she is “romantically attracted to males.” Using the answers to these two questions and the gender of the respondent, we constructed a variable with the following categories: “attracted only to opposite sex,” “attracted to both sexes,” “attracted only to same sex,” and “not attracted to any sex.” The last is *sexual behavior*, a categorical variable we built based on the number of male and female partners the respondent reported ever having sex with. The categories we created are “only opposite sex partners,” “mostly opposite sex partners” (if the proportion of opposite-sex partners is > 70% and < 100%), “both sex partners” (if the proportion of opposite-sex partners is between 30% and 70%), “mostly same-sex partners” (if the proportion of opposite-sex partners is < 30%), “only same-sex partners,” and “no sex” if the respondent has never had sex before.

To test if the relationships between sexual orientation and sleep are influenced by mental health, we included three different mental health variables: stress, depression, and anxiety. They are pre-constructed variables based on the Cohen Perceived Stress Scale (Cohen, 1994), Center for Epidemiologic Studies Depression Scale (Radloff, 1977), and Anxious Personality Scale, respectively. The scales range from 0 to 12 (stress), 0–15 (depression),^1^ and 4–20 (anxiety); the higher the score, the higher the level of reported stress, depression, or anxiety.

As controls, we included age (continuous: takes into account both year and month of birth), race/ethnicity (categorical: non-Hispanic White, non-Hispanic Black, Asian/Pacific Islander, Hispanic-any race, or other), education level (categorical: less than high school, high school, some college/vocational training, or college degree or more), in-school (dichotomous: equal to 1 if enrolled in school, 0 otherwise), self-reported health (categorical: excellent, very good, good, fair, poor), household income (categorical: $0–39,999, $40,000–74,999, $75,000 +, missing), relationship status (categorical: married, cohabiting, single/dating, or separated/divorced/widowed), and number of kids (continuous: number of biological children ever had).

### 2.2 Statistical analyses

Because of the documented gender differences in sleep behavior (Lindberg et al., 1997, Krishnan and Collop, 2006) and health-risks across sexual orientation dimensions, we conducted all analyses separately for men and women. Following descriptive statistics^2^ of the included variables, we ran multivariate logistic regressions to estimate the associations between sexual identity, sexual attraction, and sexual behavior^3^ and the outcome variables of trouble falling asleep, trouble staying asleep, and short sleep duration. Each regression included all of the aforementioned controls. Finally, we ran the models with the stress, depression, and anxiety variables included to determine if the relationships between sexual orientation dimensions and sleep changed when mental health was taken into account.

## 3 Results

### 3.1 Descriptive statistics

Table 1 depicts the descriptive statistics for the sleep, sexual orientation, and mental health variables. 6.9% of men and 8.7% of women had trouble falling asleep, while 9.9% of men and 13.8% of women had trouble staying asleep. On average, 12.3% of men slept < 6 h per night, while 7.5% of women slept < 6 h per night on a typical work day. When we look at sexual identity, we find that 93.4% of men identified as “straight,” compared to 79.6% of women. Further, 3.4% of men and 15.8% of women identified as “mostly straight.” In regards to sexual attraction, 95% of men and 89.7% of women report being attracted “only to opposite sex.” There is more variation in sexual behavior, as 89.5% of men had sex only with women, 1.9% mostly with women, 1.5% with both men and women, 1.1% mostly with men, and 1.5% only with men. While for women, 82% had sex only with men, 11.1% mostly with men, 2.3% with both men and women, 0.4% mostly with women, and 0.5% only with women. Finally, when we look at mental health, we observe that women report on average a higher score than men on each of the scales.

**Table 1:** Key variables.

	Men	Women
Sleep disturbances		
% trouble falling asleep	6.9	8.7
% trouble staying asleep	9.9	13.8
% sleeping < 6 h p. night	12.3	7.5

Sexual Identity (%)		
Straight	93.4	79.6
Mostly straight	3.4	15.8
Bisexual	0.6	2.4
Mostly gay	0.6	0.9
Gay	1.6	0.9
No specified identity	0.3	0.5

Sexual attraction (%)		
Only opposite sex	95.0	89.7
Both sexes	1.9	7.9
Only same sex	2.1	1.6
No specified attraction	1.0	0.9

Sexual behavior		
Only opposite sex partners	89.5	82.0
Mostly opposite sex partners (70–100%)	1.9	11.1
Both sex partners (30–70%)	1.5	2.3
Mostly same sex partners (70–100%)	1.1	0.4
Only same sex partners	1.5	0.5
No sex	4.6	3.7

Mental health		
Avg. anxiety scale	11.5	13.2
Avg. CESD scale	2.4	2.9
Avg. perceived stress scale	4.6	5.1
N	6688	7646

Table 2 reports the descriptive statistics for the control variables other than mental health. For both men and women, the average age of the sample is 29 years old, with the majority being white (66%). 13.2% of men and 18.6% of women are still enrolled in school, and 26.9% of men and 33.8% of women have at least a college degree. One third of the sample has an average household income lower than $40,000, while one third has higher than $75,000. Most respondents are in a co-residential union, and men have had 0.8 children on average, while women have had 1.1. Also, most individuals categorized their health as good to excellent.

**Table 2:** Control variables.

	Men	Women
Avg. age	29.0	28.7

Race-Ethnicity (%)		
White	65.8	66.0
Hispanic	12.0	11.7
Black	15.0	15.6
Asian	3.5	3.3
Other	3.6	3.4
% in School	13.2	18.6

Education level (%)		
Less than HS	10.4	7.3
High school	21.0	14.1
Some college/Vocational training	41.7	44.8
College Degree or more	26.9	33.8

Household income, $ (%)		
0–39,999	29.1	33.5
40,000–74,999	33.8	33.2
75,000 +	29.9	26.6
Missing	7.1	6.7

Relationship status (%)		
Married	36.6	43.5
Cohabiting	18.9	19.3
Separated-Divorced-Widow	6.3	7.6
Single or dating	38.1	29.7
Avg. # of children	0.8	1.1

Self-reported health (%)		
Excellent	20.6	17.7
Very good	37.7	38.9
Good	33.0	33.8
Fair	7.7	8.1
Poor	1.0	1.5
N	6688	7646

### 3.2 Multivariate regression analyses

We first ran the models without considering mental health, and the results are reported in Table 3. Starting with sexual identity, women who identify as “mostly straight” are more likely than women who identify as “straight” to report trouble falling asleep (OR = 1.52, CI: 1.24,1.87), staying asleep (OR = 1.29, CI: 1.08,1.53), and short sleep duration (OR = 1.39, CI: 1.11,1.74). Men who identify as “mostly straight” are more likely than those who identify as “straight” to report trouble staying asleep (OR = 1.62, CI: 1.08,2.42).

**Table 3:** Regression analysis of sexuality measures and sleep disturbances.

	Trouble falling asleep		Trouble staying asleep		Sleeping < 6 h	
	Men	Women	Men	Women	Men	Women
	OR/CI	OR/CI	OR/CI	OR/CI	OR/CI	OR/CI
Model 1: Sexual identity						
Straight (ref)						
Mostly straight	1.44	1.52⁎⁎⁎	1.62⁎⁎	1.29⁎⁎⁎	1.03	1.39⁎⁎⁎
	0.88,2.35	1.24,1.87	1.08,2.42	1.08,1.53	0.98,1.07	1.11,1.74
Bisexual	0.54	2.28⁎⁎⁎	0.85	1.70⁎⁎⁎	1.14⁎⁎⁎	1.02
	0.13,2.26	1.52,3.41	0.30,2.42	1.16,2.48	1.04,1.26	0.59,1.76
Mostly gay	2.17⁎	0.70	1.73	0.88	0.99	2.88⁎⁎⁎
	0.90,5.19	0.25,1.98	0.77,3.90	0.40,1.97	0.91,1.08	1.49,5.58
Gay	1.34	1.44	0.70	0.62	0.96	1.834⁎
	0.66,2.70	0.69,3.01	0.32,1.52	0.27,1.46	0.91,1.02	0.93,3.61
No specified identity	1.99	0.28	1.55	1.00	1.01	0.48
	0.56,7.11	0.04,2.11	0.44,5.39	1.00,1.00	0.87,1.15	0.11,2.02

Model 2: Sexual attraction						
Only opposite sex (ref)						
Both sexes	1.33	1.56⁎⁎⁎	2.06⁎⁎⁎	1.41⁎⁎⁎	1.92⁎⁎⁎	1.44⁎⁎
	0.72,2.47	1.20,2.03	1.29,3.30	1.12,1.77	1.24,2.98	1.07,1.93
Only same sex	1.62⁎	1.19	0.94	0.84	0.79	2.53⁎⁎⁎
	0.92,2.88	0.65,2.19	0.51,1.72	0.46,1.51	0.45,1.39	1.56,4.12
No specified attraction	1.86	0.93	1.84	0.39⁎⁎	1.24	0.48
	0.83,4.21	0.44,1.99	0.89,3.81	0.15,0.96	0.62,2.48	0.17,1.33
Model 3: Sexual behavior						
Only opposite sex partners (ref)						
Mostly opposite sex partners	0.98	1.64⁎⁎⁎	1.92⁎⁎⁎	1.51⁎⁎⁎	1.16	1.34⁎⁎
	0.48,1.99	1.30,2.05	1.18,3.15	1.24,1.84	0.67,2.00	1.03,1.73
Both sex partners	0.78	1.07	1.01	0.80	1.12	1.53⁎
	0.33,1.82	0.64,1.81	0.52,1.97	0.49,1.31	0.64,1.97	0.96,2.45
Mostly same sex partners	2.89⁎⁎⁎	1.96	1.86⁎⁎	0.44	1.36	5.25⁎⁎⁎
	1.59,5.28	0.71,5.39	1.01,3.41	0.10,1.87	0.74,2.50	2.27,12.10
Only same sex partners	0.43	2.28⁎	0.42⁎	0.93	0.55	1.73
	0.14,1.39	0.97,5.37	0.15,1.15	0.36,2.41	0.25,1.20	0.66,4.53
No sex	0.83	0.70	0.90	0.54⁎⁎	0.92	1.12
	0.50,1.37	0.41,1.18	0.58,1.38	0.33,0.87	0.64,1.33	0.72,1.73
N	6688	7646	6688	7646	6688	7646

⁎ *p* < 0.10.

⁎⁎ *p* < 0.05.

⁎⁎⁎ *p* < 0.01.

Also among women, those who identify as “bisexual” show a higher probability of having trouble falling asleep (OR = 2.28, CI: 1.52,3.41) and staying asleep (OR = 1.70, CI: 1.16,2.48) than those who identify as “straight.” Men who identify as “bisexual” are more likely to report short sleep duration than those who identify as “straight” (OR = 1.14, CI: 1.04,1.26). Further, women who identify as “mostly gay” also are more likely to report short sleep duration (OR = 2.88, CI: 1.49,5.58).

In regards to sexual attraction, women who report being romantically attracted to “both sexes” are more likely to have trouble falling asleep (OR = 1.56, CI: 1.20,2.03), staying asleep (OR = 1.41, CI: 1.12,1.77), and short sleep duration (OR = 1.44, CI: 1.07,1.93) than those who report being romantically attracted only to the “opposite sex.” Men who report being romantically attracted to “both sexes” are more likely to have trouble staying asleep (OR = 2.06,CI: 1.29,3.30) and short sleep duration (OR = 1.92, CI: 1.24,2.98) than those who report being romantically attracted only to the “opposite sex.” Women who are attracted only to the “same sex” are more likely to report short sleep duration (OR = 2.53, CI: 1.56,4.12) than those attracted only to the “opposite sex.”

Results for sexual behavior show that women who report to have had “mostly opposite sex partners” are more likely to have trouble falling asleep (OR = 1.64, CI: 1.30,2.05), staying asleep (OR = 1.51, CI: 1.24,1.84), and short sleep duration (OR = 1.34, CI: 1.03,1.73). Men who report to have had “mostly opposite sex partners” are more likely to have trouble staying asleep than those who report to have had “only opposite sex partners” (OR = 1.92, CI: 1.18,3.15). Also, men who report to have had “mostly same sex partners” are more likely to have trouble falling asleep (OR = 2.89, CI: 1.59,5.28) and staying asleep (OR = 1.86, CI: 1.01,3.41), while women who report to have had “mostly same sex partners” are more likely to report short sleep duration (OR = 5.25, CI: 2.27,12.1).

Next, we replicated the analyses with stress, depression, and anxiety included, and the results are reported in Table 4. For men regarding sexual identity, associations are no longer significant except for those who identify as “bisexual.” Men who identify as “bisexual” are more likely to report short sleep duration (OR = 2.56, CI: 1.26,5.18). Women who identify as “mostly straight” and “mostly gay” are still more likely to report short sleep duration (OR = 1.27, CI: 1.01,1.60; OR = 2.64, CI: 1.36,5.14), and women who identify as “bisexual” are still more likely to have trouble falling (OR = 1.85, CI: 1.21,2.82) and staying asleep (OR = 1.48, CI: 1.01,2.18).

**Table 4:** Regression analysis of sexuality measures and sleep disturbances, including mental health mediating variables.

	Trouble falling asleep		Trouble staying asleep		Sleeping < 6 h	
	Men	Women	Men	Women	Men	Women
	OR/CI	OR/CI	OR/CI	OR/CI	OR/CI	OR/CI
Model 1: Sexual identity						
Straight (ref)						
Mostly straight	1.15	1.24⁎	1.38	1.14	1.24	1.27⁎⁎
	0.69,1.90	1.00,1.53	0.92,2.08	0.95,1.37	0.82,1.86	1.01,1.60
Bisexual	0.46	1.85⁎⁎⁎	0.79	1.48⁎⁎	2.56⁎⁎⁎	0.92
	0.11,1.99	1.21,2.82	0.27,2.25	1.01,2.18	1.26,5.18	0.53,1.60
Mostly gay	1.73	0.58	1.49	0.80	0.91	2.64⁎⁎⁎
	0.68,4.40	0.20,1.68	0.65,3.42	0.36,1.80	0.35,2.33	1.36,5.14
Gay	1.22	1.40	0.65	0.62	0.58	1.76
	0.59,2.49	0.66,2.98	0.30,1.42	0.26,1.47	0.28,1.20	0.88,3.48
No specified identity	1.49	0.24	1.28	1.00	0.97	0.44
	0.40,5.64	0.03,1.84	0.36,4.57	1.00,1.00	0.28,3.38	0.10,1.87

Model 2: Sexual attraction						
Only opposite sex (ref)						
Both sexes	1.05	1.31⁎⁎	1.81⁎⁎	1.27⁎⁎	1.88⁎⁎⁎	1.33⁎
	0.55,2.00	1.00,1.72	1.12,2.91	1.00,1.60	1.21,2.93	0.99,1.79
Only same sex	1.44	1.13	0.86	0.83	0.80	2.42⁎⁎⁎
	0.79,2.61	0.61,2.11	0.47,1.59	0.45,1.50	0.45,1.40	1.48,3.96
No specified attraction	1.90	0.98	1.82	0.39⁎⁎	1.22	0.48
	0.83,4.35	0.45,2.11	0.87,3.80	0.15,0.97	0.61,2.45	0.17,1.33

Model 3: Sexual behavior						
Only opposite sex partners (ref)						
Mostly opposite sex partners	0.72	1.40⁎⁎⁎	1.64⁎	1.38⁎⁎⁎	1.10	1.25⁎
	0.34,1.49	1.10,1.77	0.99,2.71	1.13,1.69	0.63,1.90	0.96,1.62
Both sex partners	0.74	0.95	0.98	0.75	1.12	1.46
	0.31,1.77	0.56,1.63	0.50,1.92	0.46,1.22	0.64,1.96	0.91,2.34
Mostly same sex partners	2.28⁎⁎	1.94	1.57	0.44	1.33	4.90⁎⁎⁎
	1.21,4.31	0.69,5.46	0.84,2.92	0.10,1.89	0.72,2.45	2.10,11.46
Only same sex partners	0.42	2.40⁎	0.40⁎	0.90	0.56	1.78
	0.13,1.34	0.99,5.84	0.15,1.10	0.34,2.40	0.25,1.21	0.68,4.66
No sex	0.81	0.77	0.88	0.56⁎⁎	0.92	1.13
	0.48,1.36	0.45,1.32	0.57,1.35	0.35,0.91	0.64,1.31	0.73,1.76
N	6688	7646	6688	7646	6688	7646

⁎ p < 0.10.

⁎⁎ *p* < 0.05.

⁎⁎⁎ *p* < 0.01.

The results for sexual attraction do not change when we include the mental health variables, except for women who report to be attracted to “both sexes,” as the odds ratio for short sleep duration is no longer significant. Results for sexual behavior are no longer significant for men, except for those who have had “mostly same sex partners,” for those who have had “mostly same sex partners” still have more trouble falling asleep (OR = 2.28, CI: 1.21,4.31). Finally, the results for sexual behavior among women do not change, except for women who have had “mostly opposite sex” partners, as the odds ratio for short sleep duration is no longer significant.

## 4 Discussion

The results from our analyses confirm that sexual minorities report greater sleep disturbances than their heterosexual counterparts (Chen and Cheng-Shi, 2016), arguing that sexual minority young adults, in addition to older adults (Chen and Cheng-Shi, 2016), sleep worse than the majority. However, they also confirm the necessity to examine each sexual orientation dimension in relation to health-risks and to decompose those dimensions into more specific, intermediate categories (Lindley et al., 2012). We found considerable variation regarding sleep disturbances both across and within dimensions, thus revealing specific sexual minority groups that are at a heightened risk for reporting sleep disturbances.

Regarding sexual identity, women who identified as “mostly straight” reported more trouble falling asleep, trouble staying asleep, and short sleep duration than those who identified as “straight,” while men who identified as “mostly straight” also reported more trouble staying asleep. In addition, women who identified as “bisexual” reported having more trouble falling asleep and staying asleep, while women who identified as “mostly gay” reported more short sleep duration. Also, men who identified as “bisexual” reported more short sleep duration. However, mental health seems to partially explain the association between men who identified as “mostly straight” and trouble staying asleep, but not for men who identified as “bisexual” and short sleep duration. For women, significant associations remained for most reported identities and sleep disturbances. Mental health influencing the relationship between reported sexual identity and sleep is consistent with findings from Chen and Shiu (Chen and Cheng-Shi, 2016), as well as the gender differences.

For sexual attraction, both men and women who reported being romantically attracted to “both sexes” were more likely to have trouble staying asleep and short sleep duration than those who reported to be only attracted to “the opposite sex,” while women attracted to “both sexes” also had more trouble falling asleep. After mental health was considered, most significant associations remained. As such, a different mental health mechanism besides stress, depression, or anxiety (or a different mechanism altogether) may be at work that accounts for the undesirable sleep outcomes. For behavior, women who have had “mostly opposite sex” partners had more trouble falling asleep and short sleep duration than those who have had only “opposite sex” partners, while men who have had “mostly opposite sex” partners had more trouble staying asleep. Also, men who have had “mostly same sex” partners had more trouble falling and staying asleep, while women who slept who have had “mostly same sex” partners reported short sleep duration. Similar to identity, mental health seems to partially explain the observed relationships between sexual behavior and sleep more for men than for women.

When examining trends across and within dimensions of sexual orientation, it is clear that specific categories report greater sleep disturbances than others. In particular, men and women who identify as “bisexual,” women who identify as “mostly straight” or “mostly gay,” men and women attracted to “both sexes,” women who have had “mostly opposite sex partners” and men who have had “mostly same sex partners.” All of these categories are considered intermediate categories of sexual orientation. These results are supported by the previous finding by Lindley et al. (Lindley et al., 2012) that intermediate categories of sexual orientation are the most at-risk for various health problems. This trend points to sleep disturbances in individuals who have not settled on a specific sexual identity or on people who have greater fluidity and do not feel that standard sexual identity labels speak adequately to their experiences. As intermediate categories of sexual orientation are fairly new in research, a reconsideration of the literature regarding sexual minority health is needed. It may be the case that intermediate respondents have driven the differential levels of health issues among sexual minority populations that we have continuously seen in research. Future work is needed to understand why certain intermediate categories are consistently more at-risk. Possible explanations may focus on insecurity and instability regarding sexual orientation, which are known to cause significant strain (Ott et al., 2011, Fish and Pasley, 2015, Cass, 1979, Coleman, 1982, Everett, 2015).

Another trend is the pronounced differences between genders. Our results support past research that shows that women in general have more sleep-related complaints than men (Krishnan and Collop, 2006) and that sexual minority women sleep worse than sexual minority men (Chen and Cheng-Shi, 2016). When mental health was taken into account, most significant associations between sexual orientation measures and sleep disturbances remained for women, but not for men. Biological and genetic differences between men and women may help explain this direct association. Future research should explore potential factors, such as increased fear of discrimination, which may be related to these observed gender differences.

### 4.1 Limitations

This analysis relies solely on self-reported sexual orientation and sleep disturbance information, and self-report measures have limitations in accuracy. In particular, self-reported sleep duration may be an overestimate of sleep (Lauderdale, 2008). Self-reports can be prone to certain biases, such as social-desirability bias (Wolfson, Carskadon, Acebo, et al., 2003). However, Add Health respondents answer sensitive questions by computer (computer-assisted personal interviewing, ‘CAPI’), which may limit this. Further, most of the respondents in this sample were heterosexual, which may make it difficult to obtain unbiased and precise estimates of the odds ratios.

### 4.2 Conclusions

Overall, our findings reveal that individuals who report to be in certain intermediate categories of sexual orientation are at a heightened risk for experiencing sleep disturbances. Tailored sleep interventions may be needed to address the concerns of these specific populations, especially women within these groups. Our results confirm that neglecting all three sexual orientation dimensions, as well as the intermediate categories within those dimensions, causes us to miss important variations in health behaviors.

## Footnotes

1 Questions used for CESD Scale: In the past 7 days, how often were you “bothered by things,” “unable to shake off the blues,” “had trouble concentrating,” “felt depressed,” and “felt sad”; questions were scored 0–3 and summed for a total score

2 Descriptive statistics are weighted using Add Health's sampling weights

3 We examined correlations between dimensions of sexual orientation and found consistent results with Lindley et al. (2012), i.e. that reported identity, attraction, and behaviors can be discordant, especially among women
